# Behavioral pain scale may not be reliable in awake non-verbal intensive care patients: a case control study

**DOI:** 10.1186/s12871-024-02472-2

**Published:** 2024-02-29

**Authors:** Christian Waydhas, Christopher Ull, Oliver Cruciger, Uwe Hamsen, Thomas A. Schildhauer, Robert Gaschler, Christina Weckwerth

**Affiliations:** 1 Department of Trauma Surgery, University Hospital Essen, University Duisburg-Essen, Hufelandstraße 55, 45147 Essen, Germany; 2 Department of General and Trauma Surgery, BG University Hospital Bergmannsheil, Bürkle- de-la-Camp-Platz 1, 44789 Bochum, Germany; 3 Faculty of Psychology, FernUniversität of Hagen, Universitätsstraße 47, 58097 Hagen, Germany

**Keywords:** Pain, Non-verbal patients, Behavioral pain scale, Intensive care unit, Augmentative and alternative communication, Eye-tracking

## Abstract

**Background:**

The evaluation of pain in patients, unable of oral communication, often relies on behavioral assessment. However, some critically ill patients, while non-verbal, are awake and have some potential for self-reporting. The objective was to compare the results of a behavioral pain assessment with self-reporting in awake, non-verbal, critically ill patients unable to use low-tech augmentative and alternative communication tools.

**Methods:**

Prospective cohort study of intubated or tracheotomized adult, ventilated patients with a RASS (Richmond Agitation Sedation Scale) of -1 to + 1 and inadequate non-verbal communication skills in a surgical intensive care unit of a tertiary care university hospital. For pain assessment, the Behavioral Pain Scale (BPS) was used. Self-reporting of pain was achieved by using an eye tracking device to evaluate the Numeric Rating Scale (NRS) and the pain/discomfort item of the EuroQol EQ-5D-5 L (EQ-Pain). All measurements were taken at rest.

**Results:**

Data was collected from 75 patients. Neither the NRS nor the EQ-Pain (*r* < .15) correlated with the BPS. However, NRS and EQ-Pain were significantly correlated (*r* = .78, *p* = < 0.001), indicating the reliability of the self-reporting by these patients. Neither the duration of intubation/tracheostomy, nor cause for ICU treatment, nor BPS subcategories had an influence on these results.

**Conclusions:**

Behavioral pain assessment tools in non-verbal patients who are awake and not in delirium appear unreliable in estimating pain during rest. Before a behavioral assessment tool such as the BPS is used, the application of high-tech AACs should be strongly considered.

**Trial registration:**

German Clinical Trials Register, Registration number: DRKS00021233. Registered 23 April 2020 - Retrospectively registered, https://drks.de/search/en/trial/DRKS00021233.

## Background

Self-reporting of pain is the gold standard for monitoring and treating pain in critically ill patients. Numeric rating scales (NRS) and verbal rating scales appear best suited [1, 2]. For patients who are not able to self-report pain, behavioral assessment tools, such as the Behavioral Pain Scale (BPS), are recommended [1, 3]. Such circumstances are given when patients are (deeply) sedated, have neurotrauma, are delirious, or are otherwise in state of obtunded consciousness. Another cause of impaired communication may be an artificial airway. Patients with an endotracheal tube are always nonverbal. Also, critically ill patients with a tracheostomy tube quite often cannot communicate by speaking [4]. However, with modern concepts of no or little sedation or sedation interruption trials, they may be transiently or permanently awake [1, 5, 6]. In these patients, some augmentative and alternative communication (AAC), such as nodding/shaking head, blinking/closing eyes, and pointing with fingers, can be used to obtain a self-report on pain [2, 7]. Sometimes these variants of obtaining self-reported data are not possible due to an impaired ability to move hands or fingers. In these cases, behavioral assessment tools can be an option to assess the level of pain. In this selected group of patients, we aimed to obtain a quantitative self-report of pain using the novel AAC technology of eye tracking [8–11] and compare the results with an assessment using the BPS.

## Methods

In this prospective cohort study, we included all consecutive patients who met the following criteria: (1) endotracheal tube or tracheostomy tube and mechanical ventilation; (2) older than 18 years; (3) a score of -1, 0, or 1 point on the Richmond agitation-sedation scale (RASS) [12] and a score of less than 3 points on the nursing delirium screening scale (Nu- DESC) [13]; (4) a history of mechanical ventilation of more than 48 h; (5) expecting to be ventilated for the next 24 h; and (6) inadequate non-verbal communication skills. Inadequate non-verbal communication skills were defined as the inability to communicate sufficiently via non-tech AAC (e.g., emotions, gestures, blink, lip reading) or low-tech AAC (e.g., pen and paper, alphabet, pictures, writing boards) in the daily ICU care routine. All patients with a tracheostomy tube who were able to speak (e.g., with an unblocked cuff) were excluded.

Of 95 eligible intubated and mechanically ventilated patients, 20 patients were excluded because of the following conditions: awake but cognitively impaired (*n* = 8), extubation prior to the examination (*n* = 6), language barrier (*n* = 3), refusal to participate (*n* = 2), and sudden death before testing (*n* = 1). The remaining 75 patients were enrolled in the study.

The study was approved by the institutional review board of the Ruhr-University Bochum, Germany (18- 6620-BR) and registered at German Clinical Trials Register (DRKS00021233) on 23/04/2020. Due to internal delays the trial registration was submitted when already 11 patients had been included. Therefore, the registration had to be considered as retrospective. Informed consent was obtained by all patients, if possible, via head nodding, blinking, or the patients’ legal representatives. If necessary, appropriate consent from patients without family support was confirmed by persons unrelated to the investigation.

The manuscript adheres to the “Strengthening the Reporting of Observational Studies in Epidemiology (STROBE) statement.”

The BPS [14] was assessed in the calm and quiet patient, not having had any (painful) interventions before the examination. The BPS is a behavioral assessment tool for pain. It includes 3 items (facial expression, movements of upper limbs, compliance with the ventilator) [14]. Each item can range between 1 and 4 points, with a total score of 3 signifying no pain and a score of 12 representing the most severe pain. It has been shown to have excellent psychometric properties (e.g., internal consistency, validity, interrater reliability) in patients unable to self-report [3, 14, 15].

In all patients it was taken by the same observer (C.U.), a specialist in trauma surgery with many years of expertise in critical care, familiar with the BPS. This was followed by eye tracking (ET) intervention. It was performed by C.U. and C.We. In short, the Tobii Dynavox I-15 + eye tracking device (Tobii Dynavox, Danderyd, Sweden) was used for eye tracking. This ET consists of a monitor, cameras, projectors, and software that calculates algorithms. The projectors create a pattern of near-infrared light on the eyes. The camera takes high-resolution images of the user’s eye movements and their pattern. Machine learning, image processing, and mathematical algorithms determine the eyes’ position and gaze point on the monitor. The ET computer is a commercially available system that runs on a Windows 10 operating system. For this study, the ET was mounted on a wheel holder for bedside use in the ICU. The examination details have been outlined before [8, 16]. It has been shown that communication with intensive care patients via ET appears valid and reliable for the reporting of pain by using validated scales [9, 10, 17].

The patient’s self-reported pain was assessed with an 11-point numeric rating scale (NRS), with 0 representing “no pain” and 10 representing “worst pain imaginable.” [18]. The EQ-5D-5L is a standardized tool to measure the patients’ perception of their general health status and comprises five dimensions: “mobility,” “self-care”, “usual activities”, “pain/discomfort”, and “anxiety/depression”. Each dimension has five levels: “no problems”, “slight problems”, “moderate problems”, “severe problems”, and “extreme problems” [19]. It has been shown that the EQ-5D-5L has excellent face-validity and reliability both for the overall score and the sub dimensions (i.e. pain/discomfort) in different cultural environments and a wide range of diseases [19–21]. Here, we report on the “pain/discomfort” dimension.

Statistical analysis was performed using Microsoft Office Excel for Mac 2019 (Microsoft Corporation, Redmond, WA) and IBM SPSS Statistics Version 27 2020 (IBM Corporation, Armonk, NY). Power analysis was performed with G*Power Version 3.1 (Heinrich-Heine University of Dusseldorf, Germany) and requiring a sample size of *n* = 64 for a power of 0.8 with an alpha error of 0.05 and an average effect size of *r* = .3. Figures were created with the RStudio (version 2023.06.0). The demographic and ICU data and scores are presented as mean and standard deviation, median with quartiles, or as absolute numbers and percentages, as indicated. The t-test for independent samples was used to check whether spinal cord injured (SCI)-patients differ from non-SCI-patients. A Pearson correlation was used to check a relation between the three pain scales.

## Results

The demographic and treatment data are outlined in Table 1. It is of note that 46 patients (61.3%) were diagnosed with acute or chronic spinal cord injury, so in some of them, the BPS subscore for the upper extremity was 1 (normal) due to the type of the condition.

**Table 1:** Demographic data of the enrolled patients (*n* = 75)

Age (mean, standard deviation)	58.3 ± 17.8
Gender (f/m)	16 (21.3%) / 59 (78.7%)
Reason for ICU admission	
Major trauma	34 (45.3%)
Non-abdominal sepsis	17 (22.7%)
Acute abdomen	10 (13.3%)
Medical conditions	14 (18.7%)
Endotracheal tube / tracheostomy	11 (14.7%) / 64 (85.3%)
Duration of ventilation before examination (median, quartiles), days	15 (IQR: 8 - 25 )
Sedatives (clonidine, propofol, or isoflurane)	17 (22.7%)
Analgesia (sufentanil or piritramid)	57 (76.0%)

*Note.* The data are given as numbers (with percentages in parenthesis) or mean ± standard deviation The duration of ventilation is presented as median with interquartile range (IQR)

The mean NRS and EQ-Pain of all patients (who were at rest) were 3.95 ± 2.29 and 2.65 ± 0.97, respectively. There was no difference between the SCI and non-SCI patients, neither for the NRS (4.20 ± 2.56 vs. 3.55 ± 1.74, *p* = .24) nor the EQ-Pain (2.70 ± 1.03 vs. 2.59 ± 0.87, *p* = .64). The mean BPS of all patients was 3.25 ± 0.70 with no difference between SCI and non-SCI patients.

**Fig. 1:**
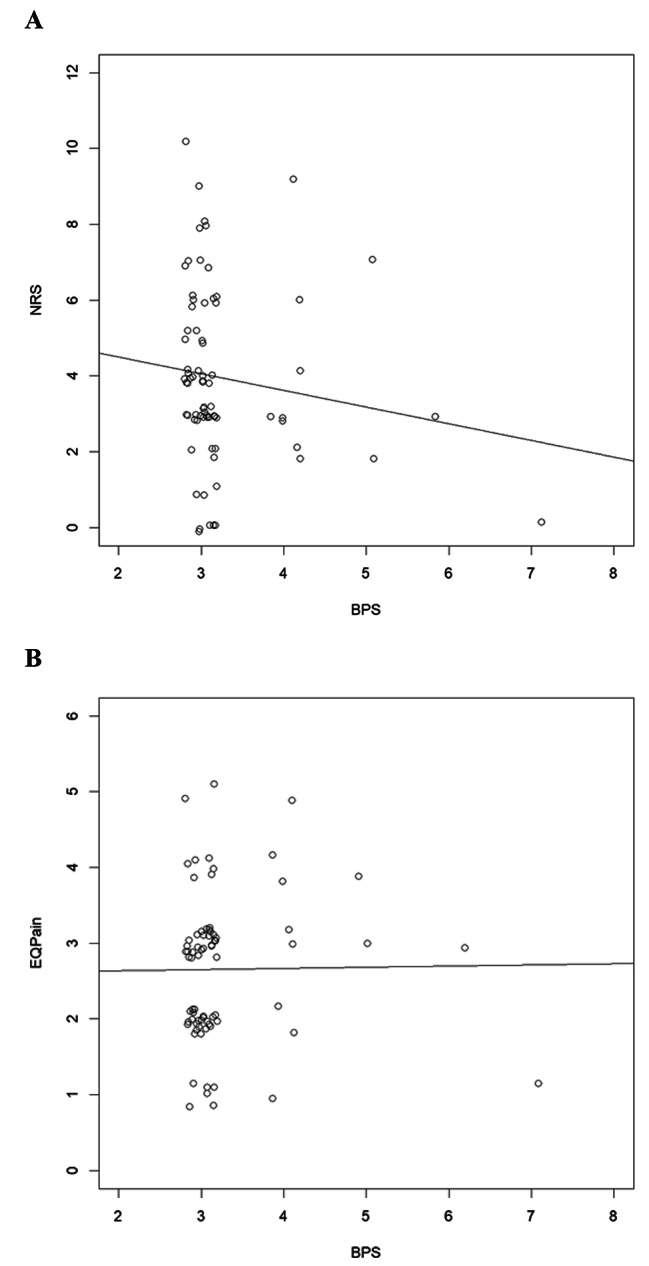
Jittered Scatterplot with trendline for the correlation between NRS and BPS (**A**) and the EQ-Pain and BPS (**B**) Note to A: NRS = Numeric Rating Scale with 0 = “no pain” and 10 = “worst pain imaginable”. BPS = Behavioral Pain Scale. Each dot represents an individual patient. Note to B: EQ-Pain = Dimension of the EQ-5D-5 L with 1 = “no problems” and 5 = “extreme Problems”. BPS = Behavioral Pain Scale. Each dot represents an individual patient.

We found no correlation between the BPS and the NRS (*r* = − .14, *p* = .25) (Fig. 1A) or the EQ-Pain (*r* = .12, *p* = .92) (Fig. 1B). This lack of correlation was true both for the SCI and non-SCI patients. Similarly, there was no correlation for any other subgroup: tracheotomized versus endotracheally intubated, duration of intubation less than 15 days versus 15 or more days, or with any of the three sub-categories of the BPS.

**Fig. 2:**
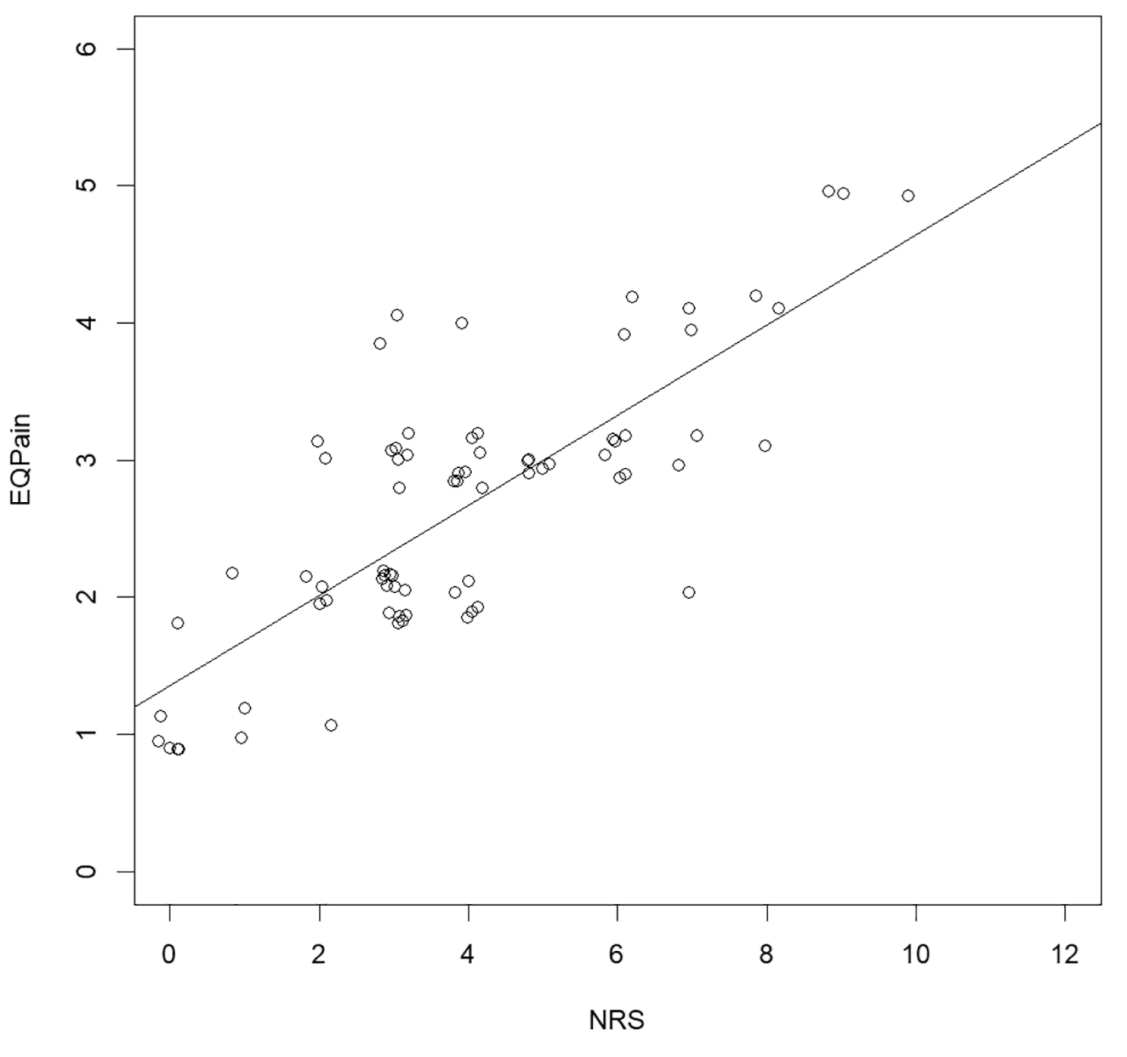
Jittered Scatterplot with trendline for the correlation between the EQ-Pain and the NRS Note: EQ-Pain = Dimension of the EQ-5D-5 L with 1 = “no problems” and 5 = “extreme problems”. NRS = Numeric Rating Scale with 0 = “no pain” and 10 = “worst pain imaginable”. Each dot represents an individual patient

In contrast, there was a significant correlation between the NRS and the EQ-Pain (*r* = .78, *p* = < 0.001) (Fig. 2).

## Discussion

Our main finding that the BPS appears to be inaccurate in many critically ill patients who are awake but unable to self-report pain with non-tech or low-tech AAC is somewhat surprising. We can rule out a falsely low BPS assessment by the observer because he is a physician experienced in intensive care and is familiar with taking the BPS. Furthermore, the BPS taken for the study coincided well with the BPS taken during routine care of the patients. Since some of the SCI patients couldn’t move their upper extremities, it couldn’t be ruled out that the BPS subcategory of “movement of the upper extremity” was systematically false (e.g., low) due to the nature of their condition. However, there was no difference in the BPS-upper-extremities between SCI and non-SCI patients (1.1 vs. 1.2). Although there could be a difference expected during painful interventions, this was not the case for our patients at rest. Furthermore, a BPS score close to 3 (no pain) has been typically reported even for surgical and post-trauma patients at rest [14, 22]. Therefore, the low BPS in our patients appears valid.

On the other hand, the self-reported results also appear valid. It has been shown that the patients were well able to give sophisticated information about their mental and psychological condition [16, 17]. On the self-esteem scale, they reported feeling trapped, not being confident, feeling frustrated, or not being understood. They also describe expectations of health improvement and experiencing good family support. Most of all, the significant correlation between two different scales of reporting pain, namely the NRS and the EQ-Pain, underscores the validity and plausibility of their self-report. In corroboration, some other studies reported a low correlation between NRS and BPS, even during painful stimuli [23].

A limitation of the study is the small sample size. However, the lack of a correlation between the BPS and self-reported pain scales is found in the overall sample as well as in sub-samples. It is unlikely that a larger number of observations would have substantially altered the results. Furthermore, we only studied the pain of patients at rest. We cannot confer that the BPS in these selected patients would also be unreliable during painful procedures and interventions. In our study group there was a large preponderance of male gender. This may be mostly explained by the high proportion of SCI and trauma patients. In severely injured patients the share of male gender was 70.1% in 2020 in Germany [24]. Another limitation could be the high portion of SCI patients. However, there was no difference in the BPS-upper-extremities between SCI and non-SCI patients, so that our results may be representative for all patients with our inclusion criteria. Last, it was a study in only a single center. Therefore, our results should be confirmed by a larger and multicenter evaluation.

## Conclusions

In conclusion, behavioral pain assessment tools in non-verbal patients who are awake and not delirious appear unreliable in estimating pain during rest. They may significantly underestimate the actual pain level and leave some patients in a painful and stressful situation. All endeavors should be made to apply non-tech and low-tech AACs to achieve a self-report of pain. Education and training of the personnel, large-scale NRS-charts, facial pain expression charts, and similar visual aids can be employed [2]. However, some critically ill patients may still be unable to reliably respond with finger-pointing due to compromised control and coordination, muscle weakness, critical illness neuro-/myopathy, paresis, and other reasons. It may also be impossible to relate an NRS of more than 5 with one hand or coordinate both hands. Before a behavioral assessment tool, such as the BPS, is used as an alternative, the application of high-tech AACs should be strongly considered. Eye tracking may offer a practical option to self-report pain in those individuals.

## Data Availability

The datasets used and/or analyzed during the current study are available from the corresponding author on reasonable request.

## Abbreviations

AAC: Augmentative and Alternative Communication
BPS: Behavioral Pain Scale
EQ-Pain: EuroQol EQ-5D-5 L
ET: Eye Tracking
ICU: Intensive Care Unit
NRS: Numeric Rating Scale
Nu-DESC: Nursing Delirium Screening Scale
RASS: Richmond Agitation Sedation Scale
SCI: Spinal Cord Injury
STROBE: Strengthening the Reporting of Observational Studies in Epidemiology
